# Consolidation With Pembrolizumab and Nab-Paclitaxel After Induction Platinum-Based Chemotherapy for Advanced Non-Small Cell Lung Cancer

**DOI:** 10.3389/fonc.2021.666691

**Published:** 2021-04-12

**Authors:** Shetal A. Patel, David E. Gerber, Allison Deal, Kathe Douglas, Chad V. Pecot, Carrie Lee, Joan Schiller, Nirav Dhruva, Jared Weiss

**Affiliations:** ^1^ Lineberger Comprehensive Cancer Center at the University of North Carolina at Chapel Hill, Chapel Hill, NC, United States; ^2^ Department of Internal Medicine, UT Southwestern Medical Center, Dallas, TX, United States; ^3^ Department of Biostatistics, Lineberger Comprehensive Cancer Center at the University of North Carolina at Chapel Hill, Chapel Hill, NC, United States; ^4^ Department of Medicine, University of Virginia, Charlottesville, VA, United States; ^5^ UNC REX Healthcare, Raleigh, NC, United States

**Keywords:** sequential therapy, PD-1 antibody, fixed duration, immune checkpoint blockade, switch maintenance

## Abstract

**Background:**

Induction with four cycles of platinum-based chemotherapy was the standard of care for metastatic non-small cell lung cancer (NSCLC) until the approval of immune checkpoint blockade (ICB) in the first-line setting. Switch maintenance therapy has shown promise in improving survival by exposing patients to novel, non-cross–resistant agents earlier in their treatment course.

**Methods:**

We performed this open-label, three-arm, randomized phase II study (NCT02684461) to evaluate three sequences of consolidation with pembrolizumab and nab-paclitaxel in patients without progressive disease post induction chemotherapy. Consolidation was either sequential with pembrolizumab for four cycles followed by nab-paclitaxel for four cycles (P→A), nab-paclitaxel followed by pembrolizumab (A→P), or concurrent nab-paclitaxel and pembrolizumab for four cycles (AP).

**Results:**

Twenty patients were randomized before the study was closed early due to the approval of first-line checkpoint inhibitors. We found that consolidation is feasible and well tolerated, with 30% of patients experiencing grade 3 toxicity. The median progression-free survival and OS in months (95% CI) in P→A were 10.1 (1.5–NR), 27.6 (1.7–NR); 8.4 (1.2–9.0), 12.7 (4.4–NR) in A→P; and 10.2 (5.1–NR), NR. Quality of life as measured by FACT-L improved in the majority of patients during the course of the study.

**Conclusion:**

Sequential and concurrent consolidation regimens are well tolerated and have encouraging overall survival in patients with metastatic NSCLC.

## Introduction

The treatment paradigm and prognosis of locally advanced and metastatic NSCLC has changed dramatically with the advent of immune checkpoint blockade using anti–PD-1/PD-L1 antibodies. Prior to the approval of these agents in the first-line setting either as monotherapy or in combination with chemotherapy, the standard treatment approach for patients with metastatic NSCLC was four cycles of platinum-based doublet chemotherapy. Studies comparing continuous versus defined duration of chemotherapy demonstrated no survival advantage for continuous treatment (1). Furthermore, there was no survival advantage for six cycles versus four cycles of induction therapy (2). Subsequently, a series of trials has evaluated the role of either “switch” or “continuation” maintenance therapy. Fidias et al. randomized patients who did not progress on gemcitabine plus carboplatin as induction therapy to either immediate consolidation with six cycles of docetaxel or treatment with docetaxel at progression. In this trial, patients randomized to the immediate docetaxel group had improved progression-free survival (PFS) and a non-statistically significant trend towards improved OS (3). Notably, OS among patients in the delayed treatment arm that received docetaxel was similar to the immediate arm, suggesting that guaranteed exposure to a non-cross–resistant treatment regimen may improve survival outcomes. In contrast, the PARAMOUNT trial examined the benefit of continuing maintenance with pemetrexed in patients with nonsquamous NSCLC treated with induction carboplatin and pemetrexed, demonstrating an improvement in PFS and OS (4, 5). Although indefinite therapy was planned, median pemetrexed administration was four cycles. The ECOG-ACRIN 5508 study evaluated pemetrexed, bevacizumab, or the combination as maintenance therapy after induction carboplatin, paclitaxel, and bevacizumab in nonsquamous NSCLC (6). Median survival was similar for the three arms, with increased toxicity noted for the combination, leading to the conclusion that single agent maintenance is efficacious. Further, ECOG-ACRIN 5508 failed to provide additional support to the hypothesis that guaranteed exposure to a novel agent is itself beneficial. As an alternative to these maintenance regimens, we treated patients with two well-tolerated non-cross–resistant therapies as consolidation after induction with platinum-based chemotherapy.

**Figure 1:**
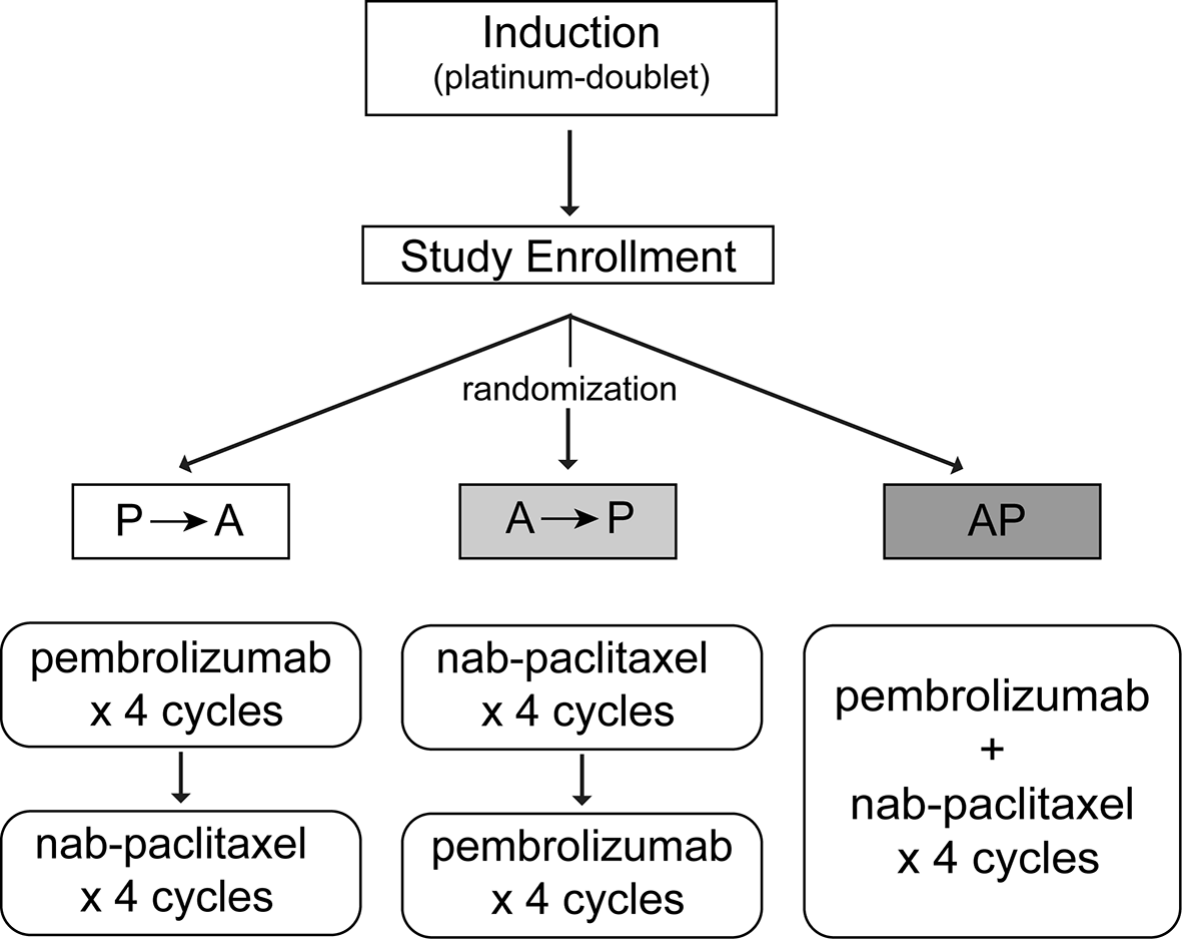
Study Schema.

Pembrolizumab is an IgG4 antibody that blocks the interaction of the T cell inhibitory receptor PD-1 with its ligands PD-L1 and PD-L2. Mechanistically, these drugs enhance anti-tumor T cell activity with durable responses seen in both squamous and nonsquamous NSCLC. At the time we designed our study, anti–PD-1 and PD-L1 antibodies were being evaluated in the second-line setting for NSCLC with significantly improved survival and tolerability compared to docetaxel (7–10). Nab-paclitaxel is a nanoalbumin bound formulation of the anti-microtubule chemotherapy paclitaxel, which has demonstrated increased response rates and a more favorable side effect profile to standard paclitaxel in advanced NSCLC patients (11). Thus, we designed our phase II study to treat patients with these two active agents after induction chemotherapy either in a sequential or concurrent fashion (**Figure 1**). Here we report survival and toxicity data for the patients on our study.

The core rationale for all three arms was that guaranteed exposure to two additional non-cross–resistant agents would improve survival. While no formal comparison was planned (or conducted) between the arms, three different sequences were evaluated in the recognition that sequence could affect efficacy. The rationale for arm A (pembrolizumab then nab-paclitaxel) was to allow for recovery of bone-marrow function and performance status with a non-cytotoxic regimen prior to additional consolidation with a cytotoxic regimen. Arm B (nab-paclitaxel then pembrolizumab) was conceived based on the hypothesis that nab-paclitaxel might further reduce the bulk of disease, resulting in superior immunotherapy efficacy. Finally, given the core hypothesis of guaranteeing exposure to additional active agents, arm C was designed to do so immediately and with the hope that there might be synergy between the agents.

## Patients and Methods

### Patients

Enrolled patients had histologically confirmed stage IV (metastatic) NSCLC and had completed four to six cycles of platinum-based chemotherapy, not including a taxane. Induction regimens were allowed to include bevacizumab, necitumumab, or cetuximab and patients were required to start treatment on protocol within 21 to 42 days of completing induction treatment. Patients had to be 18 years or older, with an Eastern Cooperative Oncology Group (ECOG) performance status of 0 to 1 and adequate hematological, renal, and hepatic organ function. Evaluable disease (by RECIST 1.1) was not required for study entry. Exclusion criteria included the use of systemic steroid therapy, immunosuppressive agents, active autoimmune disease requiring systemic treatment within the past 3 months, interstitial lung disease or pneumonitis, history of human immunodeficiency virus (HIV), hepatitis B or C or prior therapy with anti–PD-1, anti–PD-L1, anti–CD137, and anti–CTLA-4 antibodies. Patients with *EGFR* or *ALK* translocations were allowed to enroll if all approved targeted therapy options had been exhausted. This study was conducted in accordance with Good Clinical Practice guidelines and the Declaration of Helsinksi. Institutional review board approval was obtained at each investigative site.

### Study Design and Treatment Plan

We performed an open-label, three-arm, non-comparative randomized phase II study at the University of North Carolina at Chapel Hill, UNC Rex Healthcare, UT Southwestern Medical Center in Dallas, TX and Inova Schar Cancer Institute in Fairfax, VA. Intended accrual was 35 patient per arm. From August 2016 to October 2018, we screened 30 patients and 20 were randomized—seven patients each to arm A and B and six patients to Arm C (**Figure 1** **)**. Arm A consisted of sequential treatment with four cycles of pembrolizumab (200 mg every 3 weeks) followed by four cycles of nab-paclitaxel (100 mg/m^2^ on day 1, day 8 of 21-day cycle). Arm B also consisted of sequential treatment with four cycles of nab-paclitaxel followed by four cycles of pembrolizumab. Patients in Arm C received concurrent pembrolizumab and nab-paclitaxel for four cycles.

### Endpoints

The primary endpoint of the study was to estimate overall survival (OS) in each arm. Secondary objectives included estimation of PFS, objective response rate (ORR) by RECIST1.1, characterization of the toxicity profile of each arm, and description of quality of life. Overall survival is defined as the time from day 1 of treatment until death from any cause. Progression-free survival is the time from day 1 of treatment until death or progression of disease. The National Cancer Institute’s Common Terminology Criteria for Adverse Events (CTCAE, version 4.0) was used to assess toxicity. Functional assessment of Cancer Therapy-Lung questionnaires were administered on odd numbered cycles. Patient reported outcomes (PRO-CTCAE) were administered on odd-numbered cycles of both consolidations in Arms A and B and in cycles 1 and 3 of Arm C.

### Statistical Analysis

Anticipated accrual was 35 patients per arm but the study was halted early due to the approval of pembrolizumab in the first-line setting limiting enrollment. Overall survival from the first day of study treatment was estimated using the Kaplan-Meier method, separately for each arm. Progression-free survival is defined as the time from day 1 of treatment until death or first progression. All patients who received at least one dose of treatment were included in the OS and PFS estimates.

## Results

Twenty patients were randomized, with seven patients in P→A, seven patients in A→P and six patients in AP. The majority of patients enrolled in the study had adenocarcinoma histology (93%, **Table 1**). A single patient had *EGFR* mutation and had previously been treated with TKI. The median age of the patients was 63 years. Most patients had an ECOG performance status of 1 and were current or former smokers (75%). Across the three arms, 80% of patients completed protocol directed therapy, with three (15%) patients coming off treatment due to disease progression and one patient discontinuing treatment due to an adverse event. The study was halted early due to the approval of PD-1 antibodies for the first-line treatment of NSCLC, which limited accrual.

**Table 1:** Patient demographics and baseline characteristics.

Demographic	
**Gender, n (%)**	
Male	12 (60)
Female	8 (40)
**Age, years** median (min, max)	63.5 (44, 86)
**Race, n (%)**	
White	17 (85)
Black or African American	3 (15)
**ECOG PS, n (%)**	
0	5 (25)
1	15 (75)
**Smoking status, n (%)**	
Never	5 (25)
Former	12 (60)
Current	3 (15)
**Histology, n (%)**	
Adenocarcinoma	17 (94.4)
Squamous	1 (5.6)
**Mutations, n (%)**	
KRAS	4 (20)
BRAF	1 (5)
EGFR	1 (5)
KIF5B-RET	1 (5)

**Figure 2:**
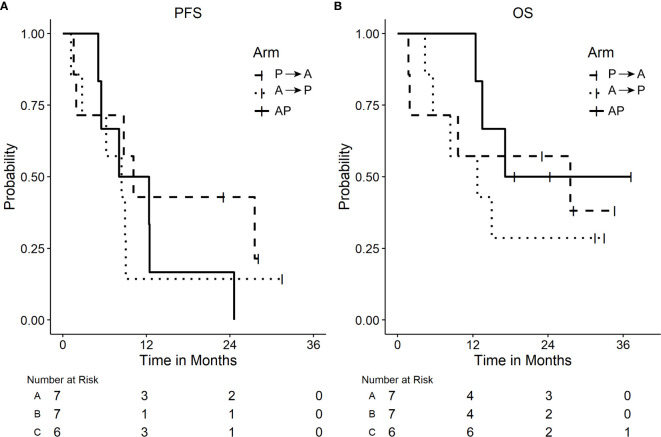
Plots of Kaplan Meier Survival curves. **(A)** Progression-free survival (PFS). **(B)** Overall survival (OS) by arm.

**Figure 3:**
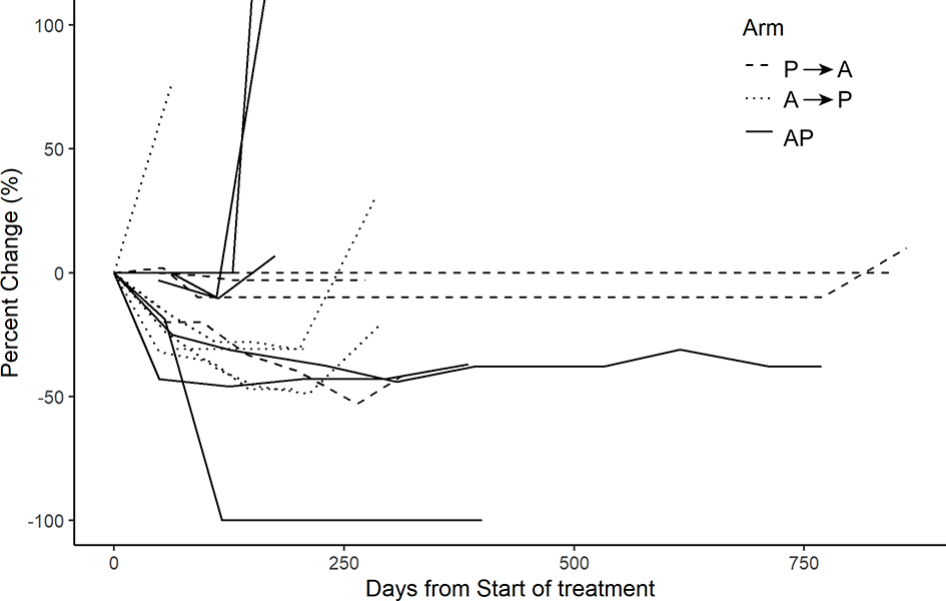
Investigator assessed change in target lesions over time (*cutoff at 100%).

Across all three arms, the median PFS (95% CI) was 8.8 months (5.1–12.5 months). The median PFS in P→A was 10.1 (1.5–NR) months, 8.4 (1.2–9.0) months in A→P, and 10.2 (5.1–NR) months in AP (**Figure 2A**). The median overall survival in P→A was 27.6 (1.7–NR) months, 12.7 (4.4–NR) months in A→P, and not reached in AP (**Figure 2B** **)**. At the time of data analysis, three patients were alive in P→A, two patients in A→P, and three patients in AP. Across all arms of treatment, 18 patients were evaluable for response, the best overall response was a complete response in two (11.1%), partial response in seven (38.9%), stable disease in seven (38.9%), and progressive disease in two (11.1%) of the patients **Figure 3**.

Treatment was well tolerated with mostly grade 1 or 2 toxicity, consistent with the known side effect profile of pembrolizumab and nab-paclitaxel (**Table 2**). Across all three treatment arms, approximately 30% of patients experienced grade 3 toxicity and no patients had grade 4 toxicity.

**Table 2:** Adverse events.

	Toxicity	Grade 1	Grade 2	Grade 3
Arm A	Creatinine increased	2		
	Dyspnea	1		1
	Adrenal insufficiency			1
	Atrial fibrillation			1
Arm B	Diarrhea	2	1	
	Alopecia	2		
	Anemia	1	1	
	Anorexia	2		
	Paresthesia	2		
	Peripheral sensory neuropathy	1	1	
	White blood cell decreased	2		
	Alkaline phosphatase increased			1
	Dehydration			1
	Neutrophil count decreased			1
Arm C	Alopecia	1	1	
	Pain	2		
	Alanine aminotransferase increased			1
	Hyperglycemia			1

Adverse events occurring in greater than two patients or any grade 3 toxicity.

**Figure 4:**
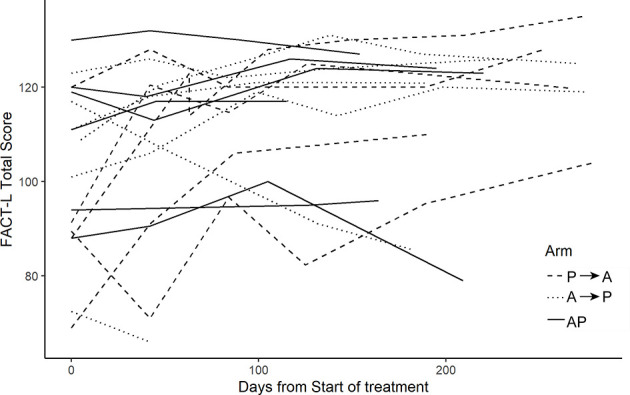
FACT-L scores. FACT-L score collected over the course of the study. Higher scores indicate improved quality of life.

**Figure 5:**
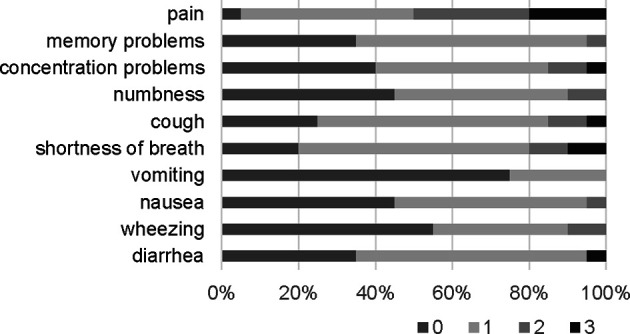
Patient reported outcomes. A composite score was generated for each symptom. The maximum score for each patient across all three treatment arms over the course of the study is summarized in the graph below.

Increased toxicity was not observed with combination therapy with nab-paclitaxel and pembrolizumab, consistent with recently reported studies of combination chemo-immunotherapy (12). Longitudinal functional assessment (FACT-L) scores improved in most patients (**Figure 4** **)**. For patient reported outcomes, a composite score ranging from 0 to 3 for each symptom was generated. The maximum score for each patient reported outcome (PRO) over the course of the study (across all three treatment arms) is summarized, again demonstrating the treatment was tolerable for the majority of patients (**Figure 5**).

Three patients with actionable oncogenes were treated on trial. One subject, with *BRAF* mutation and liver metastases was previously treated with carboplatin, pemetrexed and bevacizumab, then on arm A of this study; PFS was 8.8 months and OS was 9.6 months. Another subject who had *EGFR* and had previously been treated with TKI was treated on arm C; PFS was 5.5 months and OS 17.2 months. A final subject had *RET* fusion and was treated on arm B; PFS was 8.9 months and OS was 12.7 months.

## Discussion

Prior to the approval of immune checkpoint blockade in the first-line setting with PD-1/PD-L1 and CTLA-4 antibodies, four cycles of platinum doublet chemotherapy was a standard approach to the treatment of patients with metastatic NSCLC. In this study, we evaluated treatment with sequential or concurrent consolidation with a PD-1 antibody and nab-paclitaxel after induction. Our study was halted early due to the approval of PD-1 blockade as a first-line treatment option; however, consistent with subsequent work we found that chemoimmunotherapy is well tolerated and can generate durable disease control in a subset of patients. No new safety concerns were identified.

We chose a defined number of cycles of treatment with checkpoint blockade in contrast to continuous maintenance. This allowed patients a treatment-free interval. In the CheckMate 153 study comparing fixed 1-year to continuous treatment with nivolumab, continuous treatment was associated with improved PFS and OS (13). Patients with complete or partial responses appeared to derive greater benefit than those with stable disease. Interestingly, there was a subgroup of patients in this trial that had long-term PFS despite stopping nivolumab, similar to our study. Biomarkers that identify these long-term responders would be incredibly valuable for patient care (14).

The KEYNOTE-042 study compared chemotherapy to pembrolizumab in PD-L1 positive NSCLC patients and demonstrated superior survival with pembrolizumab given first (15). Given data on cancer evolution to evade the immune system, as well as potential immune-suppressive effects of chemotherapy, this result is unsurprising. Regardless, with this knowledge now available, we would not advocate for omission of pembrolizumab from first-line therapy in PD-L1 positive patients. In contrast, PD-L1 negative patients derive limited benefit from the addition of checkpoint inhibitors in the first-line setting, raising the question of whether sequential therapy would lead to a similar benefit. In combination therapy, which component(s) are producing efficacy and which are producing toxicity is difficult to ascertain. Sequential therapy may limit toxicity while still exposing patients to non-cross–resistant agents earlier in their treatment course. Waiting until patients symptomatically or radiographically progress may decrease the number of patients that are exposed to an alternate therapy (3). For instance, consolidation with immune stimulatory or alternate checkpoint antibodies could be examined in future studies for PD-L1 negative patients.

The impact of steroids on ICB efficacy remains controversial. While it is clear that steroids may be safely used to treat immune-related adverse events, the use of doses ≥10 mg prednisone prior to treatment may negatively impact outcomes (16). Of note for future trial combination considerations, we demonstrated that nab-paclitaxel can be safely administered without steroids, without significant nausea or vomiting.

In summary, this study closed early due to change in standard of care and with limited patient numbers no definitive conclusions may be reached. We see these results as meaningful to inform future trial considerations. More specifically, steroids may be safely omitted when nab-paclitaxel is utilized, and evaluation of sequential strategies remains reasonable within the context of carefully considered clinical trials.

## Data Availability Statement

The original contributions presented in the study are included in the article/**Supplementary Material**. Further inquiries can be directed to the corresponding author.

## Ethics Statement

The studies involving human participants were reviewed and approved by Lineberger Comprehensive Cancer Center Institutional Review Board. The patients/participants provided their written informed consent to participate in this study.

## Author Contributions

JW and AD conceived and designed the study. JW, DG, CP, CL, JS, and ND contributed to acquisition of data. JW, AD, KD, and SP analyzed and interpreted the data and drafted the manuscript. All authors contributed to the article and approved the submitted version.

## Funding

Merck and Celgene provided funding to complete this study. The funding bodies were not involved in the study design, collection, analysis, interpretation of data, the writing of this article or the decision to submit it for publication.

## Conflict of Interest

SP reports commercial research grants from Shattuck Labs and Dracen Pharmaceuticals. DG reports commercial research grants from AstraZeneca, Karyopharm, and BerGenBio, serving on a steering committee for Bristol-Myers-Squibb and is a paid consultant for Karyopharm, G1 Therapeutics, and Catalyst Therapeutics. CL is a paid consultant for Delcath Systems, Inc. JW reports commercial research grants from Merck and Celgene and has served as a paid consultant for Celgene.

The remaining authors declare that the research was conducted in the absence of any commercial or financial relationships that could be construed as a potential conflict of interest.
