# Supplementary material for: Consolidation With Pembrolizumab and Nab-Paclitaxel After Induction Platinum-Based Chemotherapy for Advanced Non-Small Cell Lung Cancer
Source: Front Oncol. 2021 Apr 12;11:666691. doi: 10.3389/fonc.2021.666691 (PMC8074674; doi:10.3389/fonc.2021.666691)
Supplement: Supplementary file 1 [file Table_1.pdf]

Supplementary Table 1: Summary of PD-L1 expression in evaluable patients

Subject	PD-L1 H score	MPS	TIL
102	25	20	2
103	200	90	1
105	0	0	3
108	1	1	1
110	25	15	1
111	0	0	1
114	1	1	1
116	275	100	1
118	20	20	1
119	5	5	2
122	0	0	2
123	15	10	2
